# Reviewing HIV-1 Gag Mutations in Protease Inhibitors Resistance: Insights for Possible Novel Gag Inhibitor Designs

**DOI:** 10.3390/molecules24183243

**Published:** 2019-09-06

**Authors:** Chinh Tran-To Su, Darius Wen-Shuo Koh, Samuel Ken-En Gan

**Affiliations:** 1 Antibody & Product Development Lab, Bioinformatics Institute, A*STAR, Singapore 138671, Singapore; 2 p53 Laboratory, A*STAR, Singapore 138648, Singapore

**Keywords:** HIV-1 Gag, Gag inhibitors, protease, protease inhibitors, drug resistance mutations, drug design

## Abstract

HIV protease inhibitors against the viral protease are often hampered by drug resistance mutations in protease and in the viral substrate Gag. To overcome this drug resistance and inhibit viral maturation, targeting Gag alongside protease rather than targeting protease alone may be more efficient. In order to successfully inhibit Gag, understanding of its drug resistance mutations and the elicited structural changes on protease binding needs to be investigated. While mutations on Gag have already been mapped to protease inhibitor resistance, there remain many mutations, particularly the non-cleavage mutations, that are not characterized. Through structural studies to unravel how Gag mutations contributes to protease drug resistance synergistically, it is thus possible to glean insights to design novel Gag inhibitors. In this review, we discuss the structural role of both novel and previously reported Gag mutations in PI resistance, and how new Gag inhibitors can be designed.

## 1. Introduction

Many anti-HIV drugs interfere directly with the viral life cycle by targeting key viral enzymes [1], e.g., reverse transcriptase inhibitors [2,3], integrase inhibitors [4,5], and protease inhibitors [6,7]. While such efforts are already hampered by the emergence of drug resistance mutations in the enzymes (e.g., in [8]), the scenario further worsens when viral enzyme substrates, such as Gag (HIV protease substrate), are found to synergistically contribute to drug resistance.

**Figure 1:**
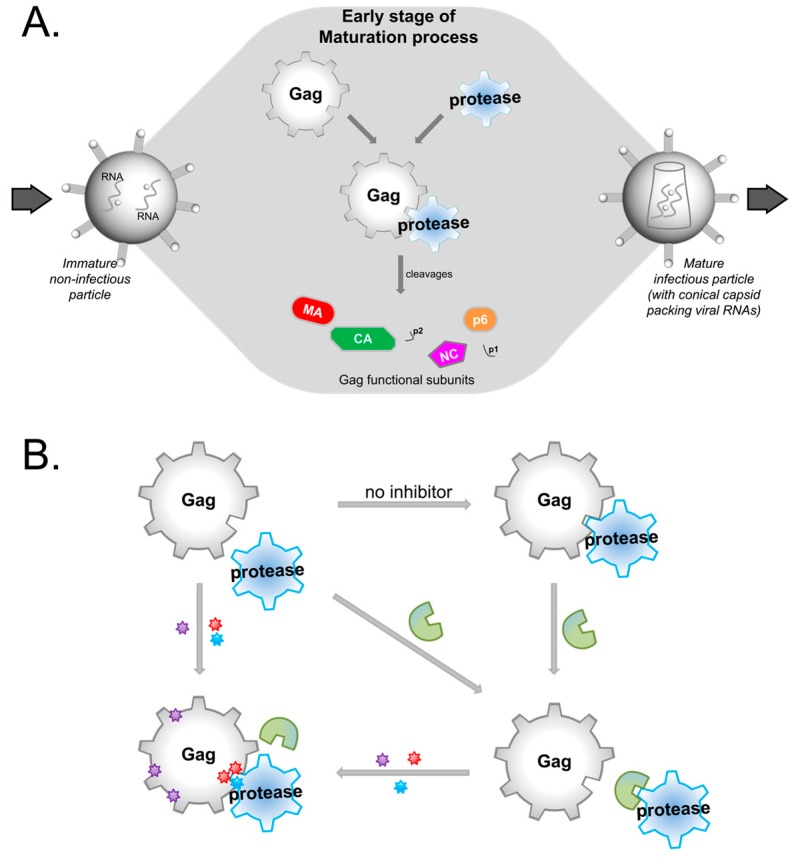
An overview of the Gag and Protease relationship. (**A**) A schematic of the early stage of viral maturation where HIV-1 Protease cleaves Gag into the functional subunits: Matrix (MA), capsid (CA), nucleocapsid (NC), p6, and two spacer peptides p1 and p2. (**B**) To inhibit viral maturation, protease inhibitors (PIs in *green*) are used to competitively inhibit protease binding of Gag. PI resistant mutations are denoted by colored stars, where those in the protease catalytic site are in *blue*, while those in Gag are *red* for cleavage sites, and *purple* for non-cleavage sites.

Gag and protease play key roles in the viral maturation process [9] where the immature HIV virion matures into the infectious virion after budding from the infected cell for the next replication cycle. Proteolysis of Gag by protease occurs during the early stage of this maturation (Figure 1A), in which the intact full length Gag precursor polyprotein is cleaved by the viral protease into functional subunits [9]. To inhibit this proteolysis, protease inhibitors (PIs) block protease activity in a competitive manner with Gag for protease binding [10].

PI-resistant mutations have been reported on Protease [11,12,13] and Gag [14,15,16,17,18] alone, or concurrently on both Protease and Gag [17,19,20,21,22], revealing an enzyme-substrate synergy to overcome PIs [23] (Figure 1B). Expectedly, Gag cleavage site mutations contribute directly to PI resistance [24], while non-cleavage site mutations contribute to drug resistance by compensating for the loss of viral fitness [22,25,26] that resulted when protease accumulates drug resistant mutations reducing its proteolytic functions.

As Gag is a larger protein than protease, and mutations (both cleavage and non-cleavage) can contribute to PI resistance, there is thus a need to study the mechanisms to how these mutations work in synergy with protease. Such studies will unravel potential weak points to which Gag can be targeted against, opening more opportunities in drug design.

## 2. Possible Targets in Gag 

The Gag polyprotein consists of components matrix (MA), capsid (CA), nucleocapsid (NC), p6, and two spacer peptides p1 and p2. The MA subunit, located at the N-terminus, is essential for targeting Gag to the cell membrane, while the CA forms a shell to protect the viral RNA genome and other core proteins during maturation. The NC is responsible for RNA packing and encapsidation [27] while the two spacer peptides p1 and p2 regulate the rate and the sequential cleavage process of Gag by protease [28]. This process of viral assembly is complemented by viral budding moderated by the small Proline-rich p6. Mutations at either the N-terminal or C-terminal of these core proteins were reported to block viral assembly and impair Gag binding to plasma membrane, thereby inhibiting viral budding [27]. 

**Figure 2:**
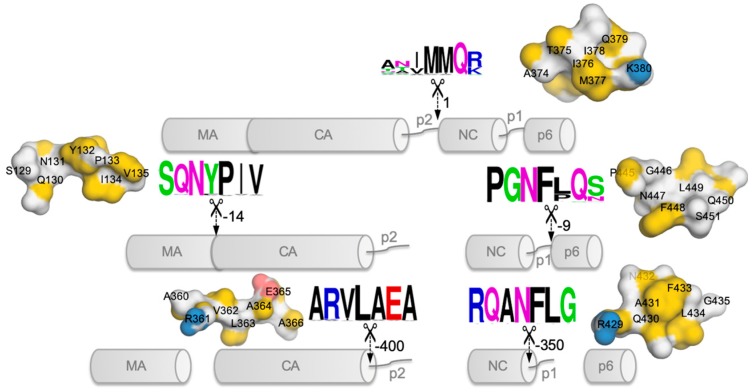
The sequential Gag proteolysis by Protease. The cleavage sites are marked by the 7-residues, along with the estimated cleavage rates [28] marked by arrows. For easy comparison, the initial cleavage site rate is set to the value of 1, while the other cleavage site values depict the reduced normalized rate. The cleavage site sequences are colored based on their physicochemical properties, e.g., hydrophobic (*black*), charged (positive: *blue*, negative: *red*), polar (other colors), and varied in text sizes based on positional conservation, using WebLogo [32,33]. Structural surface presentations of the cleavage sites are also attached for visualization.

Since the Gag cleavage sites do not share a consensus sequence (Figure 2), the recognition of the cleavage sites by protease is likely to be based on their asymmetric three-dimensional structures [29] that would fit into the substrate-binding pocket of protease [30]. The cleavage of these scissile bonds (seven-residue peptide sequences unique for each cleavage site) are highly regulated and occur at differing rates [24,28,31]. The first cleavage occurs at the site between the p2 peptide and NC domain (Figure 2), followed by the MA from CA–p2 at a rate that is ~14-fold slower than that of the first cleavage, before proceeding to release p6 from the NC-p1 domain (at a rate ~9-fold slower than the first cleavage). At the last step, the two spacer peptides p1 and p2 are cleaved from NC-p1 and CA–p2 at rates ~350-fold and ~400-fold, respectively, slower than the initial cleavage [24,28,30,31].

To date, there are nine PIs, i.e., Saquinavir (SQV), Ritonavir (RTV), Indinavir (IDV), Nelfinavir (NFV), Fos/Amprenavir (FPV/APV), Lopinavir (LPV), Atazanavir (ATV), Tipranavir (TPV), and Darunavir (DRV) in clinical treatment regimes [30]. With increasing PI resistance [34,35,36,37] and cross-resistance [21,24,35,38] conferred by protease mutations that compromise viral fitness, there is a compromise between enzymatic activity and drug inhibition by protease within its 99-residue homodimer subunits. Mapped to the resistance to several current PIs [39,40,41,42], many mutations were found to spontaneously arise as part of the natural variance [43] selected for during the treatment regimes. These mutations directly intervene with PI binding via steric perturbation at the active site, and those distant from the active site allosterically modulated protease activity [12,13,44,45,46,47,48,49,50,51,52]. However, such mutations often reduce viral fitness, resulting in future repertoires of viruses with compromised fitness [53]. This fitness trade-off is then compensated by additional mutations that restore enzymatic activity to an extent [44,48,49,54].

Reports of Gag PI-resistant mutations [17,19,20,21,22,24], whether independent or linked to protease mutations, include those that restore the reduced binding affinity to mutated proteases [17,19,20,21,22,23,24,55]. Such mutations were reported throughout the whole Gag structure with the majority on MA and p6 domains, playing a major role towards therapy failure [15,23]. In fact, multiple Gag inhibitors were rendered ineffective due to natural Gag polymorphisms [56].

New clinical protease resistant mutations are decreasingly reported, hinting a limit of the mutations tolerable within protease. On the other hand, with ~500 residues, Gag has more leeway for compensations in the Gag–protease synergy towards drug resistance. However, when compared to protease, Gag is still comparatively neglected, lacking a dedicated curated database (e.g., protease in the Los Alamos and Stanford HIV databases).

To fully study the Gag–protease synergy, there is a need to study the limitations and mechanisms by which Gag mutations arise. Although the sequencing of clinical samples is the predominant source of HIV sequences, there are attempts to study and generate novel mutations (see preprint [57] and [58,59]) for various HIV proteins. One example of such an effort [57] involved subjecting the Gag mRNA transcript to HIV reverse transcriptase (RT) to explore the repertoire of possible Gag mutations in the absence of drug or immune selection pressures. It was shown that clinically reported mutations could be generated and that the location and type of mutations incidentally avoided crucial locations and drastic changes. While such selection-free platforms can reveal the possible repertoires of Gag mutations for inhibitor design against emerging resistance, the large permutations require focusing through structural analysis for comparison to known clinical mutations.

Characterized clinical Gag mutations [17,19,20,21,22,24] are sparse, with many reported to restore reduced binding to mutated proteases [17,19,20,21,22,23,24,55]. The lack of a high-resolution structure of full-length Gag for study of these mutations makes it difficult to analyze structurally the effects of these mutations on the whole Gag during its binding to protease. Fortunately, the recent full length model of Gag [60] allowed some investigation of non-cleavage site mutations on the first cleavage site but not the subsequent sites. Nonetheless, coupling generated mutations for study in the full Gag model, it is possible to investigate the effects of the mutations before they are clinically observed. Yet, for the design of inhibitors to the remaining Gag cleavage sites or the study of the non-cleavage site mutations require structures of all the Gag subunits at every cleavage step. 

## 3. The Role of Gag Mutations in Restoring Gag–Protease Synergy in PI Resistance 

The mapping of Gag mutations associated with protease drug resistant mutations are summarized in Table 1. Gag cleavage site mutations at the p1/p6 (L449F) and NC/p1 (L449F-Q430R-A431V) sections were found to be associated to protease mutation I84V [24,61]. Similarly, Gag mutations A431V and I437V were mapped to protease mutation V82A [24,62]. Apart from compensating the loss of viral fitness, mutations P453L (Gag) and I50V (Protease) synergistically mitigated Amprenavir effectiveness (e.g., increasing IC_50_ value of Amprenavir) and Gag mutations A431V-I437V together with protease V82A were found to lead to Indinavir resistance [24].

Non-cleavage site mutations associated with PI resistance [18,22], included H219Q and R409K for Amprenavir, JE-2147, KNI-272, and UIC-94003 resistance. Gag L75R and H219Q together with Protease mutation I84V, led to Amprenavir and JE-2147 resistance. Together, these non-cleavage site mutations (synergistically with E12K, V390D, and R409K) delayed resistance to other PIs, e.g., Ritonavir and Nelfinavir [18]. Interestingly, most of these Gag non-cleavage site mutations are located on the MA–CA or p1–p6 domains. Gag MA domain mutations (e.g., R76K, Y79F, and T81A) were suggested to enhance Protease accessibility to Gag cleavage sites [15,63]. Nonetheless, the exact mechanism of such non-cleavage mutations remains elusive due to the lack of full-length Gag structure and its sequentially cleaved subunits.

**Figure 3:**
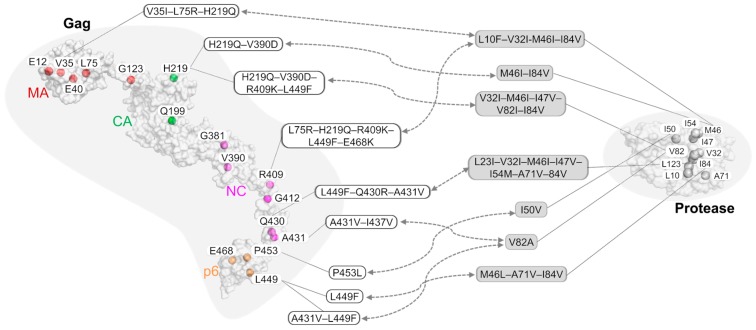
A schematic map of associated Gag–Protease drug resistant mutations. Mutation hotspots are shown on both the Gag and Protease, and representatives of paired combinations of Gag and Protease mutations are shown in boxes. More details can be found in Table 1. Gag mutations are colored according to domains: MA (red), CA (green), NC (magenta), and p6 (orange).

Limited structural research [45,47,60,64] have revealed an underlying allosteric mechanism in resistance development by Gag non-cleavage mutations that allosterically rendered the first cleavage site to be more flexible [60]. When coupled with protease mutations, several Gag compensatory mutations recovered protease binding affinities. Thus, the Gag and protease mutations synergistically formed a resistance network against multiple PIs [39,64,65]. By mapping these Gag–protease resistance relationships (Figure 3) onto our previously constructed PI cross-resistance network [64], similar combinations of Gag mutations were found to resist varied PIs, independent of their diverse chemical scaffolds [66]. 

## 4. Conceptual Novel Designs of Gag Inhibitors

Current drugs used in the ART treatment are cocktails of PIs, NRTIs, and NNRTIs [42,67,68,69]. Others treatment regimens can involve therapeutic antibodies that target viral proteins [70,71,72] or Gag epitopes [73]. Among the maturation inhibitors, Gag inhibitors have faced significant challenges. Berivimat (Panacos PA-457, Myriad MPC-4326) is the first Gag inhibitor targeting the CA–p2 cleavage site for Gag assembly [74,75,76,77,78] to have undergone clinical trials (refer to supplementary material of [56]). Yet, Gag polymorphisms [79,80] and accumulated resistant mutations during PI exposure [81,82] rendered the inhibitor candidate ineffective. Nevertheless, second-generation maturation inhibitors based on Berivimat continue to be developed and explored [83,84].

**Figure 4:**
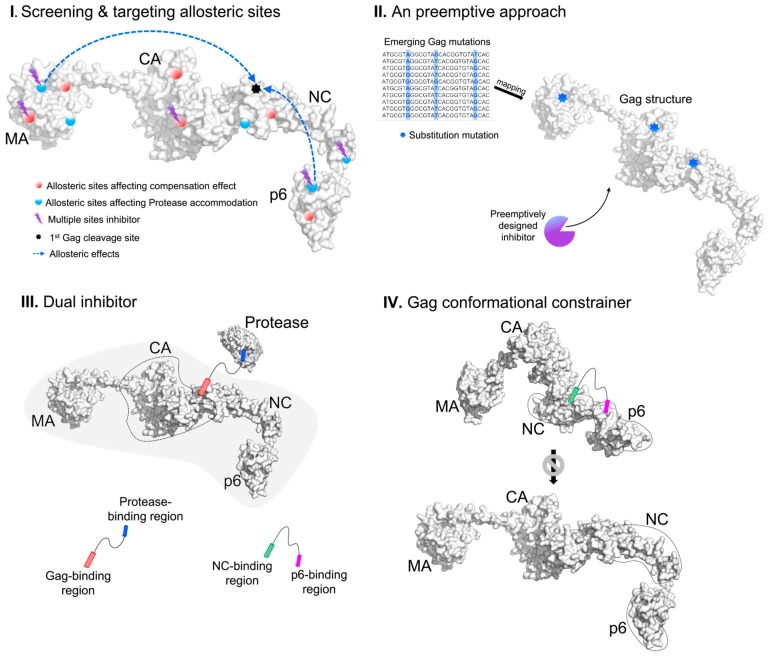
The four proposed conceptual designs of Gag inhibitors.

Due to the maturation process taking place within the closed environments inside immature virions, large biologics are less likely than small molecule inhibitors to be successful. Since Gag non-cleavage resistance mutations are found across the Gag structure (Figure 3), novel Gag inhibitors can inhibit Gag allosterically via its domain cross-talks [60,85]. With sequentially cleaved Gag structures, we propose four approaches to design Gag inhibitors (Figure 4) while keeping a holistic view of the protein as previously discussed [85].

The first approach stems from a holistic view [85] to computationally screen the whole Gag structure for novel allosteric druggable pockets to (i) inhibit compensation effects of non-cleavage mutations or (ii) directly influence cleavage sites to block protease. While the Gag cleavage sites vary in sequences, their structural similarities may allow for shared druggable allosteric inhibitors (see example of druggable allosteric pocket in HIV-1 Reverse Transcriptase [86]). Alternatively, a “multi-cleavage site” inhibitor would target the multiple cleavage sites directly (e.g., by targeting the common hydrophobic residue at the fifth position of the seven-sequence peptide, Figure 2) or indirectly via targeting multiple druggable allosteric pockets given the structural recognition. An example of such a “broad-spectrum” structural allosteric inhibitor that could inhibit HIV-1 and the Moloney murine leukemia virus (MMLV) reverse transcriptase (see preprint [87]) was found by structural similarity of the binding site. In fact, targeting multiple targets would also delay resistance since the possibility of all the target sites gaining drug resistance simultaneously is lower.

The second is a preemptive approach. By generating Gag mutations to investigate possible emerging Gag mutants (see example where Gag gene was reverse transcribed by HIV RT to generate mutations in preprint [57]), novel mutations or their combinations can be identified and modeled. Peptidomimetics in pre-emptive inhibitors of emerging resistant variants can bottleneck Gag towards eradication. Such inhibitors can include the allosteric or multiple site inhibitors, and given the wide range of possibilities, it would certainly require in-depth structural analysis to limit the permutations and combinations of mutations. This approach is easier thought than performed, given the time to model the mutations and the design of pre-emptive inhibitors that can only be tested in recombinant mutant proteins. While this approach would certainly be useful in the war against AIDS, we do acknowledge that it has a high risk of failure being dependent on theoretical mutations.

The third approach is the use of synergistic drugs targeting multiple sites that have been shown to be promising in cancer treatment, e.g., using combined biologics Trastuzumab and Pertuzumab antibodies (both of which target different epitopes on the same cancer marker Her2) with marked clinical improvements [88,89]. Further adopting from the biomimicry of bispecific antibodies that can form salt bridges between target cancer cells and the effector leukocytes for increased cytotoxicity [90], there are adoptable applications for Gag inhibitors. Chemically joining different inhibitors of (i) Gag cleavage inhibitors; (ii) Gag allosteric inhibitors (either to cleavage sites or to inhibit compensatory effects); and (iii) existing PIs can be promising modifications. Joined compounds can function as dual/triple inhibitors (Figure 4) that are more localized than separate inhibitors to the area of Gag–protease activity when already bound to a target, reducing circulation and side effects from unspecific binding. If coupled to PIs, such dual/triple inhibitors mimic antibody activity and can cause cytosolic aggregation of Gag/protease complexes for degradation or host cell death, making such combinations more promising than separate inhibitors. Since Gag inhibitors can be peptides mimicking protease binding sites to the Gag cleavage sites, it is possible to link such peptides via peptide linkers such as glycine. While linking to small molecule PIs would be more complicated, it may be possible to utilize synthetically added functional groups to the terminals of the small molecule inhibitors for directional linking. Such directional linkage would better ensure that the inhibitory groups of the small molecule inhibitors are not obscured and lose inhibitory activity. The addition of functional groups to the terminal of the small molecule inhibitors is conceptual and is highly dependent on the chemical composition of the inhibitor to also ensure that unwanted effects in terms of toxicity and stability are not introduced.

The fourth and the last approach is to disrupt Gag conformational transition during viral maturation, i.e., “throwing a wrench into a running gear” (Figure 4). Since p6 could perturb MA–CA or NC motions when Gag was compact and modulate structural stability of these Gag conformations [60], the “gear” (Gag transition conformation) could be jammed, thus destabilizing Gag assembly. Such a “wrench” could be linked with peptide inhibitors devised from the third approach of linking two inhibitors to bind at least two conserved sites in Gag to constrain Gag conformation. Potential binding sites can be the conserved regions of NC and p6 to rigidify p6 and perturb the allosteric signaling to the MA–CA region [60] to also interfere with required Gag oligomerization [91,92,93]. By preventing Gag conformational change from the compact to the extended structure, the exposure of subsequent cleavage sites could be reduced, also impairing the viral fitness by slowing down the viral maturation process.

## 5. Conclusion and Future Perspective

The increasing prominence of Gag mutations in PI resistance allows for new strategies in inhibiting HIV maturation. While there are reports of Gag resistant mutations and a full-length Gag model, much remains to be studied. There is a need to characterize Gag mutations in a curated annotated database for documenting their effects. At the same time, structures/models of Gag subunits of the various sequential cleavages are necessary for the study of documented or potential emerging Gag mutations.

Better structural and generation of emerging mutations in Gag would give rise to new types of HIV-1 drug candidates in the clinic for the next decades. These new candidates are likely to complement the current ART to form a tighter bottleneck to reduce viral load. Although it is still too early to speculate whether the Gag inhibitor candidates are likely to be small molecules or peptide inhibitors, it is reasonable to assume that they would be designed based on the structural information of the Gag cleavage sites and would primarily act to prevent protease binding or disrupt Gag assembly. Equipped with the understanding of how these non-cleavage mutations in Gag compensate viral fitness, there would be more alternative mechanisms to target for Gag inhibitors. 

## Figures and Tables

**Table 1:** Gag and Protease paired mutations compensating for viral fitness and viral replication. Gag mutations are colored according to domains: MA (red), CA (green), NC (magenta), and p6 (orange).

		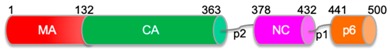	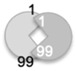
Inhibitor	Strain or Lab Clone	Mutations on Gag	Mutations on Protease
Amprenavir	HIV-1 NL4-3 (pNL4-3)	V35I–L75R–H219Q	L10F–V32I–M46I–I84V
Amprenavir	HIV-1 NL4-3 (pNL4-3)	L75R–H219Q–R409K–L449F–E468K	L10F–V32I–M46I–I84V
Amprenavir	HIV-1 NL4-3 (pNL4-3)	E12K–V35I–L75R–H219Q–V390D–R409K–L449F–E468K	L10F–V32I–M46I–I54M–A71V–I84V
JE–2147	HIV-1 NL4-3 (pNL4-3)	H219Q–V390D	M46I–I84V
JE–2147	HIV-1 NL4-3 (pNL4-3)	H219Q–V390D–R409K–L449F	V32I–M46I–I47V–V82I–I84V
KNI–272	HIV-1 NL4-3 (pNL4-3)	V35I–E40K–G123E–H219Q–G381S–R409K–A431V	V32I–M46I–A71V–V82I–I84V
UIC–94003	HIV-1 NL4-3 (pNL4-3)	E12K–E40K–G123E–Q199H–H219Q–R409K–G412D–L449F–E468K	L10F–M46I–I50V–A71V
Amprenavir	HIV-1 HXB2	P453L	I50V
BILA–1906BS	HIV-1 strain IIIB	L449F	M46L–A71V–I84V
BILA–2185BS	HIV-1 strain IIIB	L449F–Q430R–A431V	L23I–V32I–M46I–I47V–I54M–A71V–I84V
Indinavir	HIV-1 pNL4.3	A431V–I437V	V82A
Ritonavir/Saquinavir	HIV-1 subtype B ^#^	A431V–L449F	I84V

^#^ the study involves patients.
